# Effects of happy sensation training group on the symptom of anxiety, depression and sleep structure: a randomized controlled trial

**DOI:** 10.3389/fnins.2025.1414143

**Published:** 2025-02-21

**Authors:** Deping Yan, Yunqiong Wang, Jia Li, Ling Wang, Yongping Yao, Jing Deng

**Affiliations:** ^1^Sichuan Provincial Center for Mental Health, Sichuan Provincial People’s Hospital, School of Medicine, University of Electronic Science and Technology of China, Chengdu, China; ^2^Medical College, Macau University of Science and Technology, Chengdu, Sichuan, China; ^3^Institute Office, Sichuan Third People’s Hospital, Sichuan Nursing Vocational College Affiliated Hospital, Chengdu, Sichuan, China; ^4^Department of Medical, Sichuan Provincial People’s Hospital, School of Medicine, University of Electronic Science and Technology of China, Chengdu, China

**Keywords:** happiness sensation training group, chronic insomnia disorder, anxiety, depression, non-drug therapy

## Abstract

**Background:**

Non-drug therapy is a common clinical treatment for chronic insomnia, and the effect of happiness perception training group based on five senses therapy on chronic insomnia has not been evaluated.

**Objective:**

This study investigated the effects of happy sensation training group on anxiety, depression and insomnia symptoms in patients with chronic insomnia disorder.

**Methods:**

A prospective, assessor-blind, randomized controlled design was used. Adult patients with chronic insomnia were recruited from the Sleep Medicine Center of Sichuan Provincial Center for Mental Health. Participants in the training group received 10 sessions of hospital-based happy sensation training group therapy and three months of home exercises. The control group received health instruction. Results included anxiety, depression, and insomnia and were assessed at baseline, 2-week intervention, and 3-month follow-up. Polysomnography was performed at baseline and 2-week intervention. Two-way repeated measure ANOVA and Nonparametric test were used for data analysis.

**Results:**

42 patients in the training group and 43 patients in the control group accomplished 3-month follow-up. There was no significant difference in anxiety, depression, insomnia and sleep Characteristics between training group and control group at baseline (*P* > 0.05). Compared with the control group, anxiety, depression and insomnia were improved in the training group after 2 weeks and at the 3-month follow-up (*P* < 0.05), sleep latency was shortened (*P* < 0.001), and sleep efficiency, total sleep time and N2 phase sleep were increased (*P* < 0.05).

**Conclusion:**

Happiness sensation training group can effectively improve the anxiety and depression of chronic insomnia patients, so as to improve insomnia. Given its acceptability and practicality, the program could be incorporated into routine treatment.

**Clinical trial registration:**

https://www.chictr.org.cn/bin/project/edit?pid=210919, identifier ChiCTR2400082199.

## 1 Introduction

Chronic insomnia disorder (CID) is the most common type of sleep disorder. The night symptoms were mainly difficulty in falling asleep, difficulty in maintaining sleep and waking up early, while the daytime symptoms were fatigue, emotional instability and cognitive impairment. The prevalence of insomnia is about 10–20%, of which about 50% is chronic insomnia (Buysse, 2013). Sleep disorders are comorbidities that share a common pathophysiological mechanism (Riemann, 2022), so it is difficult to distinguish the causal relationship (Jihui et al., 2015). Insomnia disorders are often co-existing with anxiety, depression, impulsivity, and substance abuse (Roth et al., 2006). The comorbidities with depression are about 41% (Staner, 2010), and anxiety is about 39% (Benbir et al., 2015). Multiple studies have shown that sleep disorders may be risk factors for first-episode mental disorders, among which depression, anxiety and substance abuse are the most significant (Morin and Benca, 2012), and insomnia is an independent risk factor for first-episode depression (Blanken et al., 2020). Many studies have shown that the intervention of insomnia can also improve depressive symptoms (Blanken et al., 2019; Blom et al., 2017). Studies in cognitive neuroscience have shown that long-term insomnia will affect the prefrontal cortex of the brain’s ability to control the amygdala, resulting in impaired emotional regulation of individuals, and then produce more emotional problems, suicidal self-injury behavior and social dysfunction.

At present, the main treatment methods for insomnia include drug therapy and non-drug therapy. Drug treatment includes benzodiazepine receptor agonists, melatonin and its receptor agonists, antidepressants, antipsychotics and antihistamines, etc. These drugs have achieved good results in the treatment of acute insomnia, but their safety, tolerability and variable efficacy limit their use in the management of chronic insomnia (Cunnington et al., 2013; De Crescenzo et al., 2022; Demartinis et al., 2009). In addition, the long-term use of the above drugs will cause pathophysiological changes in sleep structure (De Mendonça et al., 2023; Paterson et al., 2009), and these changes will lead to abnormal changes in memory, respiration, gastrointestinal function, metabolic function, etc., increasing the risk of disease and death. Adverse reactions and addiction are also important reasons for restricting the clinical use of drugs and reducing the compliance of patients. Taking drugs is prone to adverse reactions such as dizziness, fatigue and sleepiness, which increases the risk of falling, and long-term use may lead to cognitive impairment, repeated insomnia and other negative effects after withdrawal (Baldwin David et al., 2013). Drug therapy has its own characteristics and advantages for the treatment of insomnia, but the current drug therapy for insomnia, especially chronic insomnia, has not reached the ideal effect.

Non-drug therapy includes cognitive behavior therapy and other psychological therapy, traditional Chinese medicine non-drug therapy and physical therapy. The American College of Physicians has identified cognitive behavioral therapy as the first-line treatment for insomnia (Koffel et al., 2018). However, due to the drawbacks of long consultation time, lack of trained providers and lack of immediate results, cognitive behavioral therapy has low treatment compliance and high shedding rate, making it difficult to carry out clinical practice (Aoki et al., 2022; Taylor et al., 2012). Traditional Chinese medicine (TCM) non-drug therapy (Chinese Academy of Chinese Medical Sciences Research Group on Clinical Practice Guidelines for Traditional Chinese Medicine for Insomnia, 2016; Liang et al., 2019) and physical therapy (Li and Xiaoli, 2022; Sulzer et al., 2013; Yao et al., 2023) are often used as clinical complementary therapy due to their great individual differences in treatment.

The five senses treatment is to stimulate people’s vision, smell, taste, hearing and touch, so that the patient’s body and mind can be relaxed, so as to achieve the purpose of treatment. Five-sense stimulation combined with horticultural therapy can improve the levels of endorphins and other hormones in depression and anxiety by stimulating the five senses, and produce a biphasic adjustment effect on the mind and body, which has been widely used in patients with chronic schizophrenia (Zhu et al., 2016) and hemodialysis (Wang et al., 2021). Happiness perception training groups are based on the five senses, through recalling or finding happiness resources around, through the five senses to re-experience or expand happiness, in order to improve mood. Studies have shown that the reason why favorite substances are lovable and produce pleasure or soothing emotions is not only due to the function of the objects themselves, but more importantly, the feelings brought to people’s five senses (Hutmacher and Schwan, 2023). In previous studies, this research group conducted an in-depth study on the mode setting and evaluation system of happy sensation training group (HSTG) (Guan et al., 2020; Wang and Jia, 2022). The Psychosomatic Medicine Center of Sichuan Provincial People’s Hospital and the University of Ulm in Germany jointly held a Sino-German psychosomatic medicine “doctor-psychology-nurse” team integrated psychological training. Based on the German “fun therapy” psychological treatment technique, combined with five perception experiences of smell, vision, taste, touch, and body movement, a HSTG with its own characteristics was formed. After the formation of the HSTG program, experts (1 chief physician of psychosomatic medicine, 2 deputy head nurses of psychosomatic medicine, and 2 psychotherapists) were organized to discuss and revise the training program to form a psychological group treatment program more suitable for Chinese patients with psychosomatic diseases. A pre-experiment was carried out in the ward of the Psychosomatic Medicine Center. According to the problems found in the pre-experiment and the feedback of the participants, it was improved and revised to form the final program. By summarizing the experience of clinical nursing staff in carrying out group psychotherapy, it was compiled into the book “Guidelines for Clinical Psychological Nursing in General Hospitals” as the guiding book for this study.

By applying HSTG to depressed patients, it is found that HSTG can significantly improve patients’ anxiety and depression, and reduce the risk of suicide (Wang et al., 2023). However, the effect of this treatment on chronic sleep associated with anxiety and depression is unclear. Therefore, this study aims to explore whether HSTG can improve sleep disorders in patients with chronic insomnia, and observe the effect of HSTG on sleep structure in patients with chronic insomnia.

## 2 Materials and methods

### 2.1 Study design and registration

This study was a prospective, assessor-blinded, two-arm randomized controlled trial. All participants provided written informed consent. This study and informed consent were both approved by the Human Research Ethics Committee of the Sichuan Provincial People’s Hospital in 2023 (Protocol NO. 2023-435). The trial was registered under Chinese Clinical Trial Registry (NO. ChiCTR2400082199).

### 2.2 Samples

In this study adult CID patients were recruited from sleep medicine center of Sichuan Provincial Center for Mental Health. Inclusion criteria for CID patients: (1) meet the diagnostic criteria for CID in DSM-5. (2) aged 18–60 years old and able to read and fill out questionnaires. (3) no serious physical disease: (4) informed consent and sign informed consent. (5) Apnea-hypopnea index (AHI) < 5. (6) Sleep latency (SL) ≥ 30 min. Exclusion criteria for CID patients: (1) patients with serious physical diseases, such as serious cardiovascular and cerebrovascular diseases, malignant tumors and other serious physical diseases. (2) patients with substance dependence or drug use history.

CID patients who met the inclusion criteria were randomly divided into intervention group and control group. CID patients who meet the inclusion criteria are assigned numbers, and a random number table is generated using an excel spreadsheet for grouping. Specific steps: Number the patients, open excel and enter the numbers in the first column; in the second column, input “=RAND (),” which will generate a random number 1. Copy this column of random number 1 (only numerical) to the third column to get random number 2; sort the random number 2 in ascending order, take the first half as the control group, and the second half as the intervention group, forming the final randomization.

The informed consent of the patients was obtained before the investigation. This study was approved by the Ethics Committee of Sichuan Provincial People’s Hospital (NO. 2023-435).

### 2.3 Intervention group

The design of the HSTG was previously described (Wang et al., 2023). The intervention included five sessions of hospital-based HSTG and 3 months of home-based practice. During hospitalization, a total of 10 sessions were performed five times a week for 2 weeks in a group psychotherapy room. The group members included a leader and assistant experienced in group psychotherapy and 8–10 inpatients. Through the exploration and experience of the five senses of smell, sight, taste, touch and body dancing, the happiness perception training group explores the happiness resources around them, so that patients can re-experience or expand happiness, thereby improving negative emotions and alleviating insomnia.

After discharge, the participants underwent home exercises for three months. Depending on the individual’s ability, we recommend practicing for 30 min at least 5 times a week. Participants also received weekly calls from interveners to provide ongoing services. Participants were encouraged to share their happy experiences during weekly telephone follow-up visits, provide timely support, and monitor their adherence to the intervention after discharge.

### 2.4 Control group

The control group received 10 health education sessions five times a week during hospitalization and telephone follow-up and health education sessions once a week after discharge for 3 months. The content of health education includes the knowledge of chronic insomnia disease and common problems, such as the risk factors of insomnia, treatment methods, sleep hygiene precautions, and the time of return visit.

### 2.5 Quality control

This intervention was provided by psychosomatic nursing staff who had undergone homogeneous training and had rich clinical experience. To ensure the quality of the group intervention, the research group members received uniform and homogenized training under the guidance of physicians and psychotherapists from the psychosomatic department. They learned the usage process and precautions of psychological assessment scales to minimize errors caused by subjective factors and ensure the accuracy of the psychological assessment scales. They also completed a full-scale assessment under their supervision. In addition, the research group members received group training, which covered a comprehensive understanding of the group program, key points and difficulties in the group leading process, and solutions to common problems. They also completed a group therapy under the supervision of an experienced group leader.

### 2.6 Measures

#### 2.6.1 General information

We used the self-designed general data scale to collect the demographic information of the participants, including age, sex, years of education, course of disease, body mass index, marital status, the only child and employment status.

#### 2.6.2 Anxiety symptoms

The severity of anxiety symptoms was assessed using the validated Chinese version of the GAD-7 scale items, which consists of 7 items, and each score ranges from 0 (not at all) to 3 (almost every day). The total score of the GAD-7 ranges from 0 to 21, with a higher score indicating more severe anxiety (Spitzer et al., 2006). The ranges of scores for severity of anxiety are as follows: no anxiety (0-4), mild anxiety (5-9), anxiety (10-13), moderate to severe anxiety (14-18), and severe anxiety (19-21). The GAD-7 Chinese version has been validated in the Chinese population with good psychometric properties (Zeng et al., 2013).

#### 2.6.3 Depressive symptoms

The severity of depressive symptoms was assessed using the validated Chinese version of the PHQ-9, which consists of nine items, with scores from 0 (“not at all”) to 3 (“almost every day”) (Kroenke et al., 2001; Spitzer et al., 1999). A higher score represents more severe depression. The ranges of scores for severity of depression are as follows: no depression (0–4), mild depression (5–9), depression (10–14), moderate to severe depression (15–19), and severe depression (20–27). The psychometric properties of the Chinese version of the PHQ-9 have been validated in Chinese populations (Wang et al., 2014; Xu et al., 2007).

#### 2.6.4 Sleep quality

The Pittsburgh Sleep Quality Index (PSQI) was used to assess participants’ sleep quality. The scale includes 7 main components, including sleep quality, the time required to fall asleep, total sleep time, sleep efficiency, sleep disorders, hypnotic drugs, and daytime dysfunction, with a total of 18 items. Total score 0–5 points: sleep quality is very good, 6–10 points: sleep quality is OK, 11–15 points: sleep quality is general, 16–21 points: sleep quality is very poor (Buysse et al., 1989). PSQI shows good psychometric characteristics with high internal consistency (Tsai et al., 2005). In our study, a score between 16 and 21 was considered to have a sleep disorder.

#### 2.6.5 Polysomnography (PSG)

Overnight PSG in bed was performed with Compumedics sleep monitoring device from 22:00 to 7:00 the next day. PSG sleep staging is based on the American Academy of Sleep Medicine Sleep and Related Events Determination Manual (version 2.6). Monitoring indicators include respiratory events: Apnea-hypopnea index (AHI), Respiratory disturbance index (RDI), Total recording time (Total recording time) TRT, Total sleep time (TST), Sleep latency (SL), percentage of sleep efficiency (TST/TRT × 100), sleep stage, blood oxygen saturation and heart rate.

#### 2.6.6 Statistical analysis

We performed statistical analysis of the data by SPSS software (version 23). Baseline features between the two groups were compared using appropriate statistical tests, including independent Student’s *t*-test, Mann-Whitney test, and Chi-square test. Two-way repeated measures ANOVA was used to determine the intervention effect, and further simple effect analysis was performed on statistically significant data. *P* < 0.05 was considered to be statistically significant.

## 3 Results

### 3.1 Patient characteristics

**FIGURE 1:**
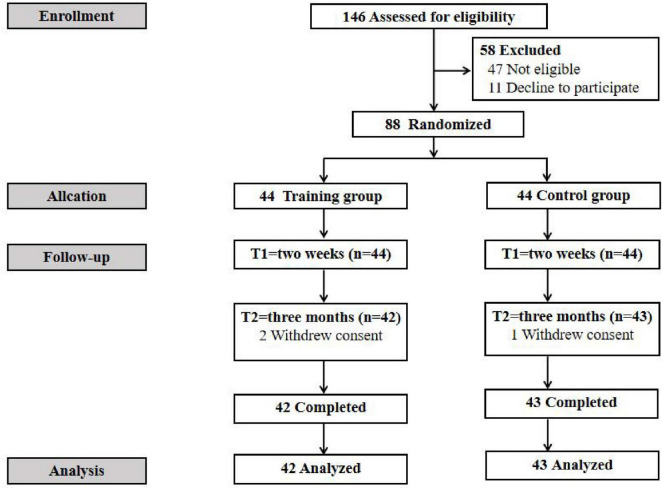
Flow diagram of participants.

A total of 146 patients were assessed for eligibility. Among these patients, 47 did not meet the inclusion criteria and 11 declined to participate. Finally, 88 eligible participants were successfully recruited. A total of 88 participants were randomly assigned to the training group and the control group, respectively (Figure 1). No statistically significant between-group differences were found in baseline characteristics (*P* > 0.05) (Table 1).

**TABLE 1:** Baseline characteristics of training and control groups.

Baseline	Training	Control	*P*-value
Number (*n*)	42	43	–
Sex (%female)	25/42	22/43	0.438
Age ($\bar{x}$ s, y)	37.98 ± 14.80	36.86 ± 14.34	0.676
Years of education ($\bar{x}$ s, y)	13.36 ± 3.17	13.17 ± 3.06	0.757
Course of disease ($\bar{x}$ s, y)	3.80 ± 3.35	4.53 ± 4.01	0.380
BMI ($\bar{x}$ s, Kg/m^2^)	23.38 ± 3.11	22.69 ± 3.01	0.306
Marital status (*n*%)			0.751
Married	20/42	19/43	
Unmarried/divorced/ widowed	22/42	24/43	
The only child (*n*%)			0.653
Yes	17/42	15/43	
No	25/42	27/43	
Employment status (*n*%)			0.058
Yes	29/42	21/43	
No/retired	13/42	22/43	

BMI, body mass index.

### 3.2 Effects on anxiety and depression

Repeated measures ANOVA showed that there was a significant interaction between group and time on anxiety and depression (*F* = 7.547, *P* = 0.001, *F* = 4.044, *P* = 0.02). Further simple effect analysis showed that there was no significant difference in anxiety and depression between training group and control group at baseline (*P* > 0.05). Anxiety significantly improved in the training group after 2 weeks (5.91 ± 0.72) and at the 3-month follow-up (2.55 ± 0.56) compared with baseline (13.05 ± 0.76), with statistical significance (*P* < 0.01 for all). Depression significantly improved in the training group after 2 weeks (8.38 ± 0.96) and at the 3-month follow-up (3.48 ± 0.66) compared with baseline (17.27 ± 0.94), with statistical significance (*P* < 0.01 for all). Participants in both groups showed a decline in anxiety and depression, but the decline was significantly smaller in the control group than that in the training group at 2 weeks (*P* < 0.001) and 3-month follow-up (*P* < 0.001) (see Table 2).

**TABLE 2:** Training effect on anxiety and depression (−x ± s, score).

Variables	Anxiety			Depression		
	**Baseline**	**2 w**	**3 m**	**Baseline**	**2 w**	**3 m**
Training	13.05 ± 0.76	5.91 ± 0.72	2.55 ± 0.56	17.27 ± 0.94	8.38 ± 0.96	3.48 ± 0.66
Control	12.67 ± 0.76	9.40 ± 0.71	5.47 ± 0.56	17.42 ± 0.93	11.72 ± 0.95	6.63 ± 0.66
*P*-value	0.729	0.01**	< 0.01**	0.906	0.016*	< 0.001***

*Marks training group compare with the control group,

*P < 0.05,

**P < 0.01,

***P < 0.001.

### 3.3 Effects on PSQI

Repeated measures ANOVA showed that there was a significant interaction between group and time on PSQI (*F* = 19.371, *P* = 0.001). Participants in both groups showed a decline in PSQI, but the decline was significantly smaller in the control group than that in the training group at 2 weeks (*P* < 0.05) and 3-month follow-up (*P* < 0.001) (see Table 3).

**TABLE 3:** Training effect on PSQI (−*x* ± *s*, score).

Variables	PSQI		
	**Baseline**	**2 w**	**3 m**
Training	17.90 ± 1.59	12.52 ± 3.09	6.93 ± 4.59
Control	17.79 ± 1.49	14.19 ± 3.45	11.72 ± 3.90
*P*-value	0.734	0.022*	< 0.001***

*Marks Training group compare with the Control group, *P < 0.05, **P < 0.001.

### 3.4 Sleep characteristics

Table 4 shows the comparison of sleep characteristics between the training and control groups. There was no significant difference in sleep characteristics at baseline in the training and control groups (*P* > 0.05). The training group showed a significantly and higher percentage sleep efficiency from baseline to T1, with differences of 72 (67, 81)% and 93 (90, 95)%, respectively. The TST, SL, REM time, percent of REM, NREM time, N2 time and percent of N2 of patients in the training group were shorter than those at baseline, with statistical significance (*P* > 0.05). In the control group, there was no significant difference in sleep characteristics except that the percent of N3 decreased compared with the baseline (*P* = 0.037).

**TABLE 4:** Comparison of sleep characteristics [M (P25, p75)].

Variables	Training (*n* = 42)				Control (*n* = 43)			
	**Baseline**	**2 w**	***Z***	***P***	**Baseline**	**2 w**	***Z***	***P***
AHI	1.2 (2.6, 3.5)	2.0 (0.6, 3.7)	0.369	0.712	2.3 (1.1, 3.9)	2.2 (1.3, 2.9)	0.350	0.726
TRT (min)	535.9 (513.7, 565.5)	524.3 (501.4, 542.9)	1.482	0.138	539.6 (513.1, 553.0)	532.2 (510.5, 550.0)	1.014	0.310
TST (min)	383.6 (342.8, 432.0)	485.0 (464.4, 507.5)	4.683	0.000	410.4 (354.0, 451.3)	404.1 (314.8, 462.7)	0.106	0.915
SL (min)	64.5 (47.3, 86.3)	11.3 (3.9, 16.6)	5.646	0.000	50.0 (36.0, 67.0)	41.0 (32.5, 54.5)	1.679	0.093
TST / TRT (%)	72 (67, 81)	93 (90, 95)	5.146	0.000	77 (69, 83)	74 (68, 87)	0.084	0.933
REM (min)	45.3 (24.8, 73.3)	72.5 (41.9, 90.1)	2.570	0.01	51.5 (25.5, 74.5)	35.0 (21.0, 53.0)	1.836	0.066
Percent of REM (%)	9.9 (4.7, 13.6)	14.4 (8.0, 17.9)	2.520	0.012	10.3 (5.0, 13.9)	7.2 (2.2, 11.4)	1.763	0.078
NREM (min)	332.8 (290.1, 377.2)	405.6 (377.3, 450.4)	4.083	0.000	361.5 (290.0, 394.5)	358.5 (254.0, 407.5)	0.145	0.885
N1 (min)	84.3 (49.9, 109.6)	63.5 (51.1, 96.8)	1.138	0.255	81 (59.9, 103.0)	78.5 (55.5, 118.5)	0.489	0.625
Percent of N1 (%)	16.7 (9.9, 21.9)	12.9 (10.2, 18.3)	1.375	0.169	16.5 (12.1, 20.6)	18.3 (12.7, 24.0)	0.769	0.442
N2 (min)	233 (193.3, 270.8)	326.2 (257.8, 360.1)	4.245	0.000	240 (171.5, 279.0)	220.5 (130.5, 276.0)	0.737	0.461
Percent of N2 (%)	48 (37.4, 56.5)	54.5 (63.4, 69.3)	4.054	0.000	48.1 (37.3, 53.9)	46.8 (35.0, 53.1)	0.737	0.461
N3 (min)	1.5 (0, 27.3)	1.3 (0, 13.6)	0.026	0.980	6.0 (0, 18.0)	3.0 (0, 34.0)	1.343	0.179
Percent of N3 (%)	0.3 (0, 5.1)	0.3 (0, 2.7)	0.077	0.939	1.4 (0, 3.8)	0.6 (0, 8.6)	2.090	0.037
Average arterial oxygen saturation	95 (93, 96)	95 (94.8, 96)	0.823	0.411	95 (94, 96)	96 (95, 97)	1.129	0.259
Average heart rate (time/min)	67.3 (63.2, 74.2)	70.0 (63.1, 77.1)	0.538	0.591	70.0 (63.2, 78.6)	68.4 (64.1, 77.0)	0.654	0.513

AHI, Apnea-hypopnea index; TRT, total recording time; TST, total sleep time; SL, sleep latency; TST/TRT, percent sleep efficiency.

## 4 Discussion

In this study, we investigated the effectiveness of HSTG in reducing the incidence of depressive mood and improving insomnia status in patients with chronic insomnia. Compared to the control group, participants who completed the happiness perception training group intervention reported a lower incidence of anxiety, depression, and insomnia to a lesser degree than before. Therefore, the group intervention of HSTG may be a convenient and effective treatment to control these symptoms simultaneously.

### 4.1 Effects on the negative affects and insomnia

Studies on the relationship between insomnia and mood (Jansson-Fröjmark and Lindblom, 2008) have found that insomnia, anxiety and depression have a two-way relationship and influence each other. Insomnia is related to emotional regulation disorders, which can lead to the imbalance of circadian rhythm, thus reducing the connectivity between the prefrontal cortex and the amygdala, resulting in emotional regulation disorders (Gruber and Cassoff, 2014). People with insomnia are 17 and 11 times more likely to develop anxiety and depression, respectively, than those without insomnia (Taylor et al., 2005). Therefore, relieving anxiety and depression will bring positive impetus to improve insomnia. Although there is ample evidence that improving insomnia symptoms can alleviate anxiety (Staines et al., 2021) and depression (Sunnhed and Jansson-Fröjmark, 2014). To our knowledge, this study is the first to demonstrate that anxiety and depression can reverse insomnia symptoms and alter sleep structure to balance circadian rhythms. This result can be partially explained by the effects of emotional regulation and social activities on various physiological pathways, including regulating endocrine homeostasis and promoting neurogenesis. Changes in these pathways may also be the underlying mechanisms by which HSTG are effective. However, further research is needed to explore the mechanism of action. Our study found that HSTG can increase patient activity engagement and improve patient adherence to treatment. In the group, participants sought out happy resources around them and shared them to improve loneliness and gain more recognition during the hospital stay. In addition to the social factor, the effect of the training is also the reason for the high attendance of the participants. In the training group, after 2 weeks of training, the detection rate of anxiety in insomnia patients decreased from 92.9 to 61.9%, the detection rate of depression decreased from 97.6 to 80.9%, and the detection rate of insomnia decreased to 28.6%.

### 4.2 Effects on the sleep structure

In terms of sleep structure, compared with healthy controls, patients with CID have disturbed sleep structure, decreased total sleep time (TST) and sleep efficiency (SE), prolonged sleep latency (SL), and decreased rapid eye movement (REM) sleep and slow-wave sleep (Reite et al., 1995). This study found that the total sleep time, sleep latency and N2 phase of the training group after the intervention of the happiness perception training group were longer than before the intervention, and the sleep efficiency was improved. In addition, the proportion of REM and REM in the training group was higher than before the intervention. Studies have shown that REM plays an important role in the regulation of overnight sleep structure and sleep neuroendocrine (Freedman et al., 2001). In the cognitive theory of insomnia (Harvey, 2002), it is also mentioned that unreasonable beliefs and excessive worry related to sleep will interfere with emotional regulation, leading to excessive emotional awakening of cognition and thus the persistence of insomnia. Nocturnal mood regulation disorders and cognitive overarousal may also alter the electrical activity of the brain during REM (Van Someren, 2021). Shortened or absent REM time and prolonged REM latency are associated with an increased risk of all-cause, cardiovascular, and other non-cancer-related death (Leary et al., 2020). Insomnia can aggravate physical disease states, worsen prognosis, hinder treatment response, and promote relapse after recovery (Chan et al., 2014).

### 4.3 Implications for nursing practice

In view of the high prevalence of chronic insomnia and its negative impact on daily life, more attention has been paid to the non-pharmacological treatment of chronic insomnia. In this study, the HSTG effectively improved insomnia by improving patients’ anxiety and depression. There is evidence that early intervention for acute insomnia can avoid progression to chronic insomnia, but because of the persistence of maintenance factors, or patients with/without anxiety depression, insomnia symptoms are repeated in patients, and eventually develop chronic insomnia. Caregivers play a key role in clinical non-drug therapy practice. Non-pharmacological treatments such as CBT-I, HSTG, and mindfulness-based stress reduction can all be done by a professionally trained caregiver. Nursing staff and patients have the longest contact time, and through inter-active learning, HSTG can be promoted throughout the hospital. It can also integrate the characteristics of different departments to improve patient compliance.

### 4.4 Limitations and future directions

In this study, we investigated the effects of HSTG on anxiety, depression and insomnia in patients with chronic insomnia, and found that both anxiety, depression and insomnia were improved. However, since the standard intervention lasted only 2 weeks, with a total of 10 times, the post-discharge intervention required patients to practice on their own accompanied by family members, and the intervention personnel only communicated and supervised by telephone follow-up. During the 3-month follow-up, the normalization and effectiveness of patients’ self-practice could not be guaranteed, resulting in the continuity of treatment could not be effectively guaranteed. In addition, the age range of the included people in this study is 18–65 years old, with a wide age range, and the types of insomnia problems faced by patients of different ages and the incidence of anxiety and depression are also different. Therefore, in the follow-up study, the research team will narrow the age range of included patients to ensure that patients of the same age can participate in the study and improve the accuracy of the study. In terms of intervention, intervention videos can be recorded to establish participants’ completion day records to monitor participants’ completion and improve compliance. Establish a doctor-patient sharing group, and encourage participants to share their practice experience in the wechat group, and provide timely support to solve problems related to the practice.

## 5 Conclusion

This randomized clinical trial found that HSTG is an effective and safe non-pharmacological treatment for anxiety, depressive mood and insomnia in patients with chronic insomnia. Our findings constitute subjective and objective evidence of the efficacy and safety of HSTG to treat comorbidity anxiety, depression, and insomnia.

## Data Availability

The raw data supporting the conclusions of this article will be made available by the authors, without undue reservation.
